# Acute Respiratory Viral Infections Among Adult Patients in Edirne, Turkey

**DOI:** 10.3390/tropicalmed10020058

**Published:** 2025-02-19

**Authors:** Sebnem Bukavaz, Kultural Gungor, Merve Köle, Galip Ekuklu

**Affiliations:** 1 Health and Vocational College, Trakya University, 22030 Edirne, Turkey; 2 Department of Infectious Diseases and Clinical Microbiology, Kırklareli University, 39100 Kırklareli, Turkey; k_cultural@hotmail.com; 3 Department of Medical Microbiology, Edirne Sultan 1. Murat State Hospital, 22030 Edirne, Turkey; dr.mervekole@gmail.com; 4 Department of Public Health, Faculty of Medicine, Trakya University, 22030 Edirne, Turkey; ekuklu@hotmail.com

**Keywords:** acute respiratory viruses, COVID-19, influenza, RSV, multiplex PCR

## Abstract

**Background/Objectives:** This study aimed to evaluate the prevalence of viral agents identified by Multiplex PCR in acute respiratory viral infection (ARVI) patients at Edirne Sultan 1, Murat State Hospital, from April 2023 to April 2024, and to investigate the relationship between monthly average humidity and viral positivity rates. **Methods:** The study included 764 adult patients (aged 18 and older) diagnosed with influenza symptoms. Respiratory viral samples were collected and analyzed for COVID-19, influenza A and B, and RSV using Multiplex PCR, with results evaluated retrospectively. Continuous variables in the study were compared using a *t*-test, and categorical variables were compared with a chi-square test. A logistic regression analysis was performed for the analysis of COVID-19. In this analysis, PCR positivity was the dependent variable, while age, gender, and humidity level served as independent variables. **Results:** COVID-19 PCR positivity was detected in 142 patients (18.6%), with INF-A (influenza A) in 13 (3.7%), INF-B (influenza B) in 15 (4.2%), and RSV in 2 (0.6%). Higher humidity (over 60%) was associated with reduced viral PCR positivity rates for COVID-19 and influenza B, while low (up to 40%)/normal (40–60%) humidity correlated with positivity rate (*p* < 0.05 for both). Logistic regression analysis indicated that high humidity levels offer protection against COVID-19 (OR: 0.356; 95% CI: 0.245–0.518). **Conclusions:** Our study provides essential epidemiological data by summarizing monthly virus distribution in Edirne.

## 1. Introduction

Environmental parameters and human behavior significantly influence the seasonality of acute respiratory viral infections (ARVIs). Research indicates that temperature and humidity affect the stability and transmission rates of respiratory viruses. Additionally, environmental factors impact the host’s immune responses—both congenital and adaptive—to viral infections in the respiratory tract. Other contributing factors include seasonal variations in absolute humidity, sunlight exposure, vitamin levels, and patterns of human interaction, which influence the contact rates between infected and susceptible individuals [1]. Previous studies, particularly one on droplet size and Influenza survival, indicate that higher temperatures and absolute humidity reduce the virus’s survival rate within an hour [2,3]. The effect of humidity on ARVIs varies by virus type, but numerous studies indicate that high humidity helps reduce the spread of COVID-19 and Influenza. For instance, enveloped viruses like influenza, measles, and SARS-CoV-2 persist longer in low relative humidity (30%), while non-enveloped viruses such as adenovirus and rhinovirus thrive in high humidity (70–90%) [4]. Low relative humidity (RH) increases the generation of infectious virus-laden aerosols exhaled from infected people [5]. Furthermore, low humidity leads to a greater strain on the respiratory tract to moisten the inhaled air, and the drying out of parts of the respiratory mucosa increases the chance of entry of pathogens. Low humidity also increases the impact of particulate matter on the respiratory tract, which is associated with an increased risk of infection [6]. In a study conducted for COVID-19 in Turkey, it was reported that higher temperatures and higher relative humidity (e.g., 38 °C and 95% relative humidity) eliminated virus viability [7].

ARVIs are prevalent and can lead to significant morbidity and mortality, particularly in young children, the elderly, and immunocompromised adults [8,9,10]. The most common viruses detected in ARVIs are influenza virus (INF), human rhinovirus (HRV), respiratory syncytial virus (RSV), human coronavirus (HCoV), and parainfluenza virus (PIV) [11]. Understanding the epidemiology and seasonality of these infections is crucial for planning vaccination and treatment strategies [12].

In recent years, the Multiplex polymerase chain reaction (PCR) method has been developed to simultaneously detect all respiratory viruses responsible for infections using a single sample and test, complementing other microbiological diagnostic methods [13]. PCR offers several advantages over traditional culture and direct fluorescence antibody (DFA) tests: it provides faster results, identifies sub-serotypes of certain viruses, detects viruses that cannot be cultured, and demonstrates higher sensitivity and specificity [14,15,16]. This study aimed to determine the frequency of viral agents identified through Multiplex PCR in patients diagnosed with an ARVI at Edirne Sultan 1, Murat State Hospital, from April 2023 to April 2024. It also sought to examine the relationship between the monthly average humidity and the positive rates of viral pathogens in Edirne.

## 2. Materials and Methods

This study, conducted from 1 April 2023 to 1 April 2024, examined adults (over 18 years) who visited the outpatient clinics and emergency department of Edirne Sultan 1, Murat State Hospital, with flu symptoms were diagnosed based on WHO criteria, which include fever over 38 °C, weakness, rhinitis, headache, sore throat, cough, myalgia, and other related symptoms in hospital patients. PCR tests were conducted, and those with confirmed positive results were diagnosed with ARVI (acute respiratory viral infection). All ARVI patients displayed flu-like symptoms, and those with flu symptoms and positive PCR results were classified as having ARVI. Patients were included in the study regardless of chronic disease or vaccination status.

Nasopharyngeal swabs were used to obtain respiratory smears from patients with suspected viral respiratory tract infections and sent to the routine microbiology laboratory of the hospital for Multiplex PCR testing; influenza A and B and RSV results were retrospectively evaluated. Humidity data were grouped into three categories: low (up to 40%), normal (40–60%), and high (over 60%) [5]. Humidity values were averaged retrospectively from the diagnosis, considering the viruses’ incubation periods, with data collected monthly from the Provincial Directorate of Meteorology.

This study received ethical approval from the Trakya University Faculty of Medicine Non-Interventional Scientific Research Ethics Committee under decision no. 09/26 on 6 May 2024.

### 2.1. Respiratory Tract Multiplex PCR

The respiratory samples (nasopharyngeal swabs) obtained from the patients with respiratory infections were transported to the Medical Microbiology Laboratory under cold chain protocols using a viral transport medium (Tüseb, Diakit, Istanbul, Turkey). RNA isolation was performed with a Tüseb vNAT viral extraction kit (Diakit, Turkey). Quantitative PCR (qPCR) was conducted on the extracted samples using a Tüseb Diakit Multiplex -SARS-CoV-2/Influenza A&B/RSV RT-qPCR kit (Diakit, Turkey) on a Bio-Rad CFX 96 Real-Time Detection System (Bio-Rad, Hercules, CA, USA). Each viral agent was classified as positive or negative based on the amplification curves and cycle threshold (Ct) values according to the kit’s instructions.

The PCR assay was performed on a fully automated, labor-saving machine that was effective in reducing subjective operation errors. The PCR assays used are CE (Conformité Européenne), ISO 9001 (International Standart of Organization) certified, and approved by the National Department of Health.

### 2.2. Statistical Analysis

A statistical analysis was conducted using SPSS version 22. Continuous variables were expressed as mean ± SD and categorial variables as *n* (%). Continuous variables in the study were compared using a *t*-test, and categorical variables were compared with a chi-square test. A logistic regression analysis was performed for the analysis of COVID-19. In this analysis, PCR positivity was the dependent variable, while age, gender, and humidity level served as independent variables. Due to initially unknown variables and a large dataset of 764 patients, we selected the Forward Selection model as the most suitable approach, given its low Bayesian Information Criterion (BIC) value.

In the forward logistic regression model, all independent variables were initially included. Unrelated variables were excluded at each step. To analyze the relationship between PCR results and daily average relative humidity, we compared the daily average relative humidity in the PCR (+) and PCR (−) groups with a *t*-test based on data from the Edirne Meteorology Directorate covering 1 April 2023 to 1 April 2024. This included 14 days for COVID-19, 3 days for INF-A and INF-B, and 4 days for RSV, in accordance with incubation periods. A *p*-value of <0.05 was deemed significant.

## 3. Results

### 3.1. Virus Positivity

This study evaluated a total of 764 patient samples, consisting of 400 female samples (52%) and 364 male samples (47%), all of which were respiratory swabs. COVID-19 PCR positivity was detected in 142 patients (18.6%), with INF-A in 13 (3.7%), INF-B in 15 (4.2%), and RSV in 2. A gender-based analysis of COVID-19 positivity revealed that 70 females (17.5%) and 72 males (19.8%) tested positive. INF-A positivity was observed in 10 females (5.2%) and 3 males (1.9%), while INF-B positivity was observed in 11 females (5.7%) and 4 males (2.5%), and RSV positivity was detected in 1 female and 1 male. No statistically significant difference was found between genders for the viruses studied (Table 1).

In analyzing the relationship between PCR positivity and age within the viral panel, the 142 COVID-19-positive patients had a mean age of 42.0 ± 16.7 years. For the 13 INF-A-positive patients, the mean age was 40.5 ± 18.2 years, while the 15 INF-B-positive patients had a mean age of 43.3 ± 20.9 years. The age of the two RSV-positive patients was 88 and 34 years. No significant relationship was found between virus positivity and age (*p* > 0.05).

The laboratory findings revealed leukopenia, leukocytosis, lymphopenia, lymphocytosis, and elevated C-reactive protein (CRP) levels. Among the 764 patients, leukopenia was present in 14 (1.8%), 10 (14%) of COVID-19 positives, 3 (10%) of InfA/B positive and 1 (0.2%) of non-ARVI. Leukocytosis was present in 95 (12.4%), 4 (3%) of COVID-19 positives, 2 (7%)of Inf A/B positives, and 87 (14.6%) of non-ARVIs. Lymphopenia was present in 56 (7.3%), 7 (5%) of COVID-19 positives, 1 (3.5%) of Inf A/B positives, and 48 (8%) of non-ARVIs. Lymphocytosis was present in 1 of non-ARVIs (0.1%). Elevated CRP levels were observed in 72 patients (9.4%): 27 (19%) of COVID-19 positives, 3 (11%) of Inf A/B positives, and 42 (7%) of non-ARVIs. No significant relationship was found between COVID-19 positivity and CRP levels, although lower leukocyte (*p* > 0.05) and lymphocyte counts (*p* > 0.05) were noted (Table 2).

Of the patients, 73 (9.6%) developed pneumonia; 8 (5.6%) of COVID-19 positives, 1 (3.5)% of Inf A/B positives and 64 (10.8%) of non-ARVIs, with 38 (52%) hospitalized in services and 11 (1.4%) requiring intensive care. Among the hospitalized patients, six tested positive for COVID-19, and one tested positive for RSV. Sixteen patients (2.1%) received oseltamivir: 3 (2.1%) with COVID-19, 3 (10%) with InfA/B, and 10 (1.6%) among non-ARVIs. In addition, 119 patients (15.6%) were treated with antibiotics: 4 (2.8%) of COVID-19 positives, 2 (7%) of InfA/B positives, and 113 (19%) of non-ARVIs. Increased CRP levels and antibiotic treatment were reported in 8 COVID-19 positive patients who developed pneumonia. There were six patient deaths (0.8%), including one COVID-19-positive individual. Pneumonia, treatment, and death rates are detailed in Table 2.

#### Seasonal Impact

Regarding seasonal effects, patient presentation dates revealed a total of 764 hospital visits: 240 (31.4%) in autumn, 239 (31.3%) in winter, 231 (30.2%) in spring, and 54 (7.1%) in summer. Statistically, autumn, winter, and spring had similar application numbers, with no significant differences, while summer showed a significant decrease, dropping by more than fourfold. An analysis of viral PCR positivity indicated that 70 of the 142 COVID-19 patients were positive in autumn (49%; September 19%, October 25%, and November 7%); 32 in winter (22%; December 6%, January 11%, and February 5%); and 35 (24%) in spring (3%; July 1% and August 2%). A statistical relationship was found between the rise in COVID-19 cases and the autumn season (*p* < 0.05).

Regarding INF-A, 13 positive patients were recorded: 5 in autumn (38%; September 30% and October 8%), 0 in winter, 7 in spring (53%; March 7% and April 46%), and 1 in summer (7%; June). The increase in INF-A cases during autumn and spring was statistically significant (*p* < 0.05). Regarding INF-B, 15 positive patients were recorded: 7 in autumn and spring (53%; March 7% and April 46%); 0 in winter; and, like INF-A, 1 in summer (7%). PCR positivity was also statistically significant in autumn and spring (*p* < 0.05). Regarding RSV, two positive patients were recorded, both in winter (December and January); however, due to the small sample size, a statistical analysis was not conducted (Table 3).

**Figure 1:**
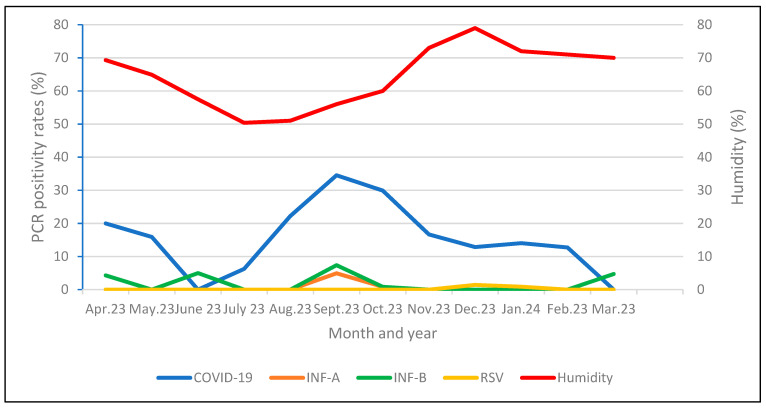
Distribution of viral PCR positivity rates according to months and monthly average humidity.

To examine the relationship between the average relative humidity and viral PCR positivity, daily humidity data were averaged over 14 days for COVID-19, 3 days for INF-A and INF-B, and 4 days for RSV, focusing on the dates when patients tested positive for viruses. COVID-19 positivity was higher at normal humidity levels, with 54.2% (77 out of 142 patients), and lower at high humidity, showing 45.8% (65 out of 142 patients). The INFA was detected in five patients (38.5%) at normal humidity and eight patients (61.5%) at high humidity. The INFB was positive in seven patients (46.7%) at normal humidity and eight patients (53.3%) at high humidity. The differences were statistically insignificant (*p* > 0.05) (Table 3). When comparing viral PCR positivity rates with the monthly average humidity, a higher humidity correlated with fewer cases, particularly for COVID-19 and Inf B, while a lower average humidity coincided with peak cases (*p* < 0.05 for COVID-19 and Inf-B) (Figure 1).

Figure 1 shows the average humidity and positivity rates for COVID-19, INF-A, INF-B, and RSV by month.

PCR positivity was taken as the dependent variable, and age, gender, and humidity level were taken as independent variables. In the analysis conducted with the logistic regression model, it was found that high humidity levels are associated with protection against COVID-19 (OR: 0.356; Cl 95%: 0.245–0.518) (Table 4).

## 4. Discussion

Respiratory infections exhibit cyclical patterns every one to two years, indicating that their seasonal distribution is influenced by population sensitivity and environmental factors affecting virus survival, transmission, and behavior, which fluctuate throughout the year [2]. Recent studies have proposed three mechanisms to explain the seasonality of epidemic respiratory viruses in humans: (i) virus stability and transmissibility under varying environmental conditions, particularly humidity and temperature; (ii) human behavior, including indoor/outdoor activities, crowding, and holiday travel; and (iii) the effect of changing environmental conditions on host defense mechanisms [1]. The relative contributions of these factors to respiratory virus seasonality remain unclear [17]. Due to the challenge of developing lasting immunity after respiratory infections, populations often remain susceptible, leading to intensified transmission in densely populated areas and resulting in epidemics. The timing of the flu season, typically from October to February or March, varies each year. From an epidemiological perspective, the WHO and European countries monitor these viruses. Data from the European Respiratory Viruses Surveillance Summary (ERVISS) are analyzed by selecting the four weeks with the highest number of reported cases, and the average and standard deviation are calculated.

Our study analyzed the data obtained from Edirne Sultan 1, Murat State Hospital, and compared them with data from a second-level healthcare provider in ERVISS.

The results indicate that, for COVID-19, the average positive rate is 19% (SD: 0.96) over four weeks, rising to 36% (SD: 0.96) in the first week and dropping to 35% (SD: 0.57) in the subsequent four weeks. ERVISS reports similar results for first-level health service providers at 21% [18].

Influenza A and B were assessed together, showing an average positivity rate of 30% (SD: 3.37) for the fourth week of 2024 and 43% (SD: 2.06) across the following four weeks. According to the WHO data, RSV rates peak at week 48, with a 4-week average of 18% (SD: 0.72) from this week, while our results show only 1% (SD: 0.15) for the same period (excluding children).

Our COVID-19 and influenza A/B positivity rates are approximately 1.8 and 1.4 times higher than in European countries, respectively. Factors contributing to this include a lack of respiratory virus surveillance in healthcare institutions and insufficient differentiation among primary healthcare providers for upper respiratory infections. RSV rates are reported to be 18 times higher in European countries, although, in Turkey, RSV is noted as the most common upper respiratory agent in children [19]. Since children were excluded from our study, our RSV rates appear lower in comparison. Furthermore, while ERVISS aggregates diagnostic results from European nations, the specific diagnostic methods are not detailed. Nonetheless, Multiplex PCR is the most widely used respiratory virus panel globally [20].

The evaluated viral panel showed no significant differences in age or gender among patients. While RSV and influenza virus infections were noted in children in previous studies, our focus on adults revealed no differences in gender or virus incidence [21]. In terms of laboratory and clinical findings, our results indicate that 19% of COVID-19-positive patients had elevated CRP levels, particularly in severe cases associated with pneumonia. However, only 5.6% of these patients developed pneumonia, suggesting that other cases were likely due to bacterial infections.

Research on the seasonal distribution of respiratory virus infections has expanded. Over the years, different virus panels have dominated during seasonal shifts. A 24-year retrospective study examining the impact of climate change on virus positivity found that RSV and influenza viruses predominantly occur from November to April, with negligible activity in the summer [22,23].

Our study shows a 43% increase in influenza virus applications compared with April. The total number of viruses in our region decreased in winter, resulting in infrequent influenza cases during that season. Consistent with the existing literature, only one positive case was reported in June during the summer months. In the case of Inf-B, positivity rates decline with rising humidity, but the low positivity rate in our study makes it challenging to link this directly to moisture levels. Research on the effects of relative and absolute humidity on virus transmission indicates that the mechanisms connecting vapor pressure (VP) and influenza virus survival time (IVS) are similar to those associated with relative humidity. These include (1) a heightened production of virus-laden droplet nuclei under low-VP conditions and (2) increased IVS in low-VP environments [24,25]. Even in the controlled environment of an animal shelter (22.2 °C, 50% humidity, and a regulated light/dark cycle), the transmission rate of influenza virus was observed to be higher in winter (November–April, 58.2%) than in summer, despite using the same viral load in experiments [1]. The relationship between high humidity values and COVID-19 protection necessitates the evaluation of other environmental factors, such as sunlight and wind. The decrease in COVID-19 in the summer months may also be related to the UV exposure of the sun [26]. In addition, the effect of humidity values, spending too much time indoors, indoor humidity values, and hygiene should also be considered in the transmission of disease in winter. Furthermore, the interplay between temperature and humidity can influence the viability of the virus in the environment. Warmer temperatures, often associated with higher humidity, may reduce the stability of the virus on surfaces and in the air, thereby decreasing transmission rates [27].

A study conducted across 21 countries revealed that the seasonal patterns of coronaviruses resemble those of influenza and RSV in temperate regions. Heat maps indicate that most cases in the northern hemisphere occur between December and March, while in the southern hemisphere, they peak from July to September. The human coronavirus 229E was found to survive the longest in stabilized aerosols at 4 °C, with relative humidity levels of 30%, 50%, or 80% having minimal impact. It survives better in 80% humidity at 30 °C and in 50% humidity at 20 °C. Our study noted that, during the peak COVID-19 period, when relative humidity was between 50 and 55%, it reached 80% from December to February, coinciding with a decline in cases to 10% or lower. Furthermore, an analysis of the human coronavirus in relation to environmental factors indicated that relative humidity, along with UV radiation and heat, significantly influences virus survival [2]. Upper respiratory tract viruses, particularly influenza and RSV, are most prevalent in urban areas, tropical regions, and cold climates in North America and Europe. Their impact is exacerbated in areas with weak health systems and low vaccination rates. However, there are significant evidence gaps, especially from Central Asia, North and Central Africa, and South America. In Africa, research has predominantly focused on vector-borne diseases and public health systems, overlooking the potential effects of climate change on maternal and child health, respiratory infections, and malnutrition [28].

Our study faced a restriction due to insufficient data on the specific criteria for requiring only the COVID-19 test and the four-virus panel test (COVID-19, influenza A/B, and RSV) for patients presenting flu symptoms at the clinic. The clinician’s judgment influenced this choice, preventing standardization. Furthermore, the absence of data regarding patients’ chronic illnesses obscured the determination of the relationship between hospitalization, death in intensive care, and viral infections. Evaluating only humidity among environmental factors restricted the comparison with others. The study hypothesis should be tested in a multicenter study covering different regions with different seasonal climate patterns.

## 5. Conclusions

In our study, we found that respiratory virus positivity in Edirne over one year does not vary by demographic characteristics. Additionally, corona, influenza, and RSV viruses are more prevalent in certain months, and high relative humidity may provide protection against corona and RSV infections, aligning with the existing literature. Further research on virus transmissibility and the impact of humidity and environmental factors is needed.

## Figures and Tables

**Table 1:** Frequency distribution of detected viral pathogens by gender.

Viruses	Female (*n* = 400) *n* (%)	Male (*n* = 364) *n* (%)	Total (*n* = 764) *n* (%)
COVID-19	70 (17.5%)	72 (19.8%)	142 (18.6%)
INF-A	10 (5.2%)	3 (1.9%)	13 (3.7%)
INF-B	11 (5.7%)	4 (2.5%)	15 (4.2%)
RSV	1 (0.3%)	1 (0.3%)	2 (0.3%)

Table 1 displays the percentages of women and men and the overall total for positive cases of COVID-19, INF-A, INF-B, and RSV. COVID-19: human Coronavirus; INF-A: influenza A virus; INF-B: influenza B virus; RSV: respiratory syncytial virus. Distribution did not significantly differ between males and females (*p* > 0.05).

**Table 2:** Clinical and laboratory Findings.

**Lab. Find.**	**Leukop.**	**%**	**Leuko.**	**%**	**Lymp.**	**%**	**Lymph.**	**%**	**CRP** **Eleva.**	**%**	**Clin. Find.**	**Pneumo.**	**%**	**Treat.**				**Stay** **Hosp./ICU**	**%** **(Total)**	**Death**	**%**
														**Ost.**	**%**	**Ant.**	**%**				
***n* = 764**	14	1.8	95	12.4	56	7.3	1	0.1	72	9.4		73	9.6	16	2.1	119	15.6	38/11	6.1	6	0.8
**COVID-19** ***n* = 142**	10	14	4	3	7	5	1	0.7	27	19		8	5.6	3	2.1	4	2.8	5/1	4.2	1	0.7
**Influenza** **(INF-A + INF-B)** ***n* = 28**	3	10	2	7	1	3.5	0	0	3	11		1	3,5	3	10	2	7	0/0	0	0	0
**Non ARVI cases** ***n* = 592**	1	0.2	87	14.6	48	8	0	0	42	7		64	10.8	10	1.6	113	19	33/10	6.9	5	0.8

Table 2 shows laboratory and clinical findings, hospital/ICU stay, and death percentage for all admitted patients. Laboratory (Lab.) and clinical (Clin.) findings were not significant (*p* > 0.05). Leukop: leucopenia; leuko: leukocytosis; lymp: lymphopenia; lymph: lymphocytosis; CRP eleva: CRP elevation; pneumo: pneumonia; treat: treatment; Ost: oseltamivir; Ant: antibiotic; Stay Hosp/ICU: hospital/intensive care unit.

**Table 3:** Distribution of viral PCR positivity according to months.

Months	Number of Tested Samples *n* (%) *	Relative Humidity %	COVID-19 *n* (%) *	Inf-A *n* (%) *	Inf-B *n* (%) *	RSV **
April 2023	140 (18)	69.3	28 (20)	6 (43)	6 (43)	0
May 2023	44 (6)	64.9	7 (16)	0	0	0
June 2023	20 (3)	57.5	0	1 (5)	1 (5)	0
July 2023	16 (2)	50.4	1 (6)	0	0	0
August 2023	18 (2)	51.0	4 (22)	0	0	0
September 2023	81 (11)	56.0	28 (35)	4 (5)	6 (7)	0
October 2023	117 (15)	60.0	35 (30)	1 (0.9)	1 (0.9)	
November 2023	42 (6)	73.0	7 (17)	0	0	0
December 2023	70 (9)	79.0	9 (13)	0	0	1
January 2024	114 (15)	72.0	16 (14)	0	0	1
February 2024	55 (7)	71.0	7 (13)	0	0	0
March 2024	47 (6)	70.0	0	1 (0.9)	1 (0.9)	0
Total	764		142	13	15	2

In Table 3, the number of patients admitted to the hospital by month, the average monthly humidity, and positivity rates for COVID-19, INF-A, INF-B, and RSV are presented. * Proportion of the admitted patients to the hospital by month and proportion of the total number of cases for COVID-19, Inf-A and Inf-B. ** No statistical analysis was performed due to the fact that there were only 2 cases.

**Table 4:** Results of multiple logistic regression of COVID-19 positivity on gender, age, and high average humidity level 14 days before diagnosis.

	*p*	OR	95% CI	
			Lower	Upper
Age		1.007	0.996	1.018
Gender Men	0.657	0.919	0.632	1.335
High humidity	<0.001	0.356	0.245	0.518

Table 4 shows a multivariate analysis of virus positivity with age, gender, and high average humidity level 14 days before diagnosis. OR: odds ratio; CI: confidence interval.

## Data Availability

All data generated or analyzed during this study are included in this article.
